# Dose-Dependent Hepatotoxicity of Diethyl Phthalate in Female Wistar Rats

**DOI:** 10.3390/toxics14020174

**Published:** 2026-02-16

**Authors:** Mehmet Cihan Yavaş, Gül Şahika Gökdemir, Kübra Tuğçe Kalkan, Salih Varol, Fazile Cantürk Tan

**Affiliations:** 1 Department of Biophysics, Faculty of Medicine, Mardin Artuklu University, 47200 Mardin, Turkey; 2 Department of Physiology, Faculty of Medicine, Mardin Artuklu University, 47200 Mardin, Turkey; gulsahikagokdemir@artuklu.edu.tr; 3 Department of Histology & Embryology, Faculty of Medicine, Kırşehir Ahi Evran University, 40100 Kırşehir, Turkey; tugce.kalkan@ahievran.edu.tr; 4 Department of Biophysics, Faculty of Medicine, Erciyes University, 38039 Kayseri, Turkey; salihvarol@erciyes.edu.tr (S.V.); fcanturk@erciyes.edu.tr (F.C.T.)

**Keywords:** diethyl phthalate, liver inflammation, hepatotoxicity, genotoxicity, rat

## Abstract

Phthalates are a class of compounds commonly used as plasticizers in various industrial and consumer products. In line with the increasing environmental and biological exposure concerns regarding these compounds, this study investigated the dose-dependent effects of diethyl phthalate (DEP) on the liver in a subacute rat model. Diethyl phthalate (DEP) was given orally by gavage to female Wistar albino rats at doses of 100, 300, and 600 mg/kg body weight per day for 21 days in order to assess liver tissue and associated function test levels. Liver function was evaluated by analyzing serum biochemical data. Liver tissues were evaluated using histopathological staining (H&E and Masson’s trichrome staining), immunohistochemical analysis of IL-1β and TGF-β, tissue ELISA for IL-6 and TNF-α, and comet assay to determine DNA damage. DEP exposure was found to cause significant, dose-dependent histopathological changes in liver tissue, including hepatocyte necrosis, cytoplasmic vacuolization, sinusoidal dilation, and vascular congestion. AST levels were significantly increased compared to the control group, while no significant changes were observed in other serum biochemical parameters. Compared to the control group, the expression of pro-inflammatory cytokines (IL-6 and TNF-α), IL-1β, and TGF-β was found to be elevated in the DEP-treated groups, and their levels increased with increasing exposure dose. DEP exposure also caused significant DNA damage in liver tissue. These findings indicate that despite an increase in AST levels observed in subacute DEP exposure, there were limited changes in serum biochemical parameters; serum liver enzymes alone may not fully reflect the extent of hepatic damage, and DEP can cause significant inflammatory, histopathological, and genotoxic effects in liver tissue.

## 1. Introduction

Phthalate esters are chemical additives that are frequently used to improve the flexibility and durability of plastic materials in a range of consumer and industrial applications [1,2,3]. The fact that these compounds do not chemically bond to the polymer matrix causes them to migrate over time into food, environmental surfaces, and biological tissues [4,5]. The frequent occurrence of phthalate metabolites in human populations indicates continuous and unavoidable exposure [3,6]. Toxic effects of the broad phthalate group have been reported to include increased oxidative stress, inflammatory cytokine production, disruption of lipid metabolism, and genotoxic effects [1,3,6,7]. Therefore, phthalates are considered among environmental toxins that particularly target the liver [1,2,8].

The liver is central to phthalate toxicity because it is responsible for the metabolism of lipophilic foreign chemicals [1,2]. Experimental studies with various types of phthalates have reported hepatocellular degeneration, peroxisome proliferation, fatty degeneration, sinusoidal dilation, and necrotic foci [1,2,9]. Additionally, it has been demonstrated that phthalates activate inflammation-regulating signaling pathways such as NF-κB, MAPK, JNK, and STAT, which in turn increase pro-inflammatory cytokines such as tumor necrosis factor alpha, interleukin-6 (IL-6) and interleukin-1 beta (IL-1β) [3,10]. Increased oxidative stress, glutathione depletion, increased MDA, and mitochondrial dysfunction are other common mechanisms of phthalate hepatotoxicity [1,3,7]. Additionally, DNA strand breaks, 8-OHdG enhancement, and genetic damage confirmed by the comet assay are also significant toxicity components of phthalates [1,6,7].

Within this broad toxicological framework, diethyl phthalate (DEP) is classified among low molecular weight phthalates and is widely used, particularly in cosmetics, detergents, air fresheners, and coating materials [4,5]. The routine detection of DEP metabolites in human urine indicates widespread and regular exposure [6,8]. Although DEP was considered “relatively low toxic” for many years, research in the last decade has shown otherwise; it has been clearly demonstrated that DEP can produce toxic effects at both cellular and systemic levels [4,5,8,11].

Significant hepatotoxicity has been reported in experimental animal studies with DEP. Chronic DEP administration (200 mg/kg, 30 days) in rats has been shown to lead to significant increases in serum AST, ALT, ALP, and GGT levels, impaired lipid metabolism, and hepatocyte degeneration [4]. In a study of long-term DEP administration, increases in ALT/AST levels, triglycerides, hepatocyte vacuolization, and fatty changes were reported in Wistar rats [9,11]. DEP exposure has also been reported to impair lipoprotein function, leading to fatty liver-like changes and hypolipidemia [8]. These findings indicate that DEP directly interferes with lipid metabolism.

The effects of DEP on the inflammatory response are also becoming increasingly better understood. According to a recent study, DEP significantly elevated oxidative stress, activated JNK and STAT signaling pathways, and enhanced the production of IL-1β, IL-6, TNF-α, iNOS, and COX-2 in macrophages [10]. DEP exposure leads to increased ROS production and nitrosative stress, contributing to the progression of inflammation and cellular damage at the tissue level [3,10]. These mechanisms are consistent with histopathological findings in the liver, such as portal inflammation, sinusoidal dilation, and cellular infiltration [4,9,11].

Previous studies have detailed how phthalates, particularly DEHP, contribute to collagen deposition and fibrosis development by activating fibrogenic pathways such as TGF-β1, Smad, NF-κB, and p38 MAPK [1,12]. While data directly investigating tissue fibrosis for DEP are limited, biochemical evidence suggests that similar mechanistic pathways may be activated [8,10,11]. In our study, the use of Masson’s trichrome staining will scientifically strengthen the evaluation of collagen deposition and fibrogenic activity.

The genotoxic effects of DEP exposure have also been confirmed by numerous studies. Both in animal studies and cell culture models, as well as in human urine samples, DEP has been shown to cause DNA breakage, oxidative DNA damage, increased ROS, and comet assay positivity [1,6,7,13]. The observation of oxidative DNA damage, even with repeated low-dose exposures, supports the genotoxic potential of DEP [7,10,13].

However, the scarcity of studies in the current literature that simultaneously evaluate DEP exposure in a dose-dependent manner using histopathological, biochemical, inflammatory, genotoxic, and fibrogenic parameters indicates a significant knowledge gap [1,5]. Accordingly, investigating the exposure to different doses of DEP in a female Wistar albino rats model using comprehensive parameters such as H&E and Masson Trichrome staining, serum biochemical analyses, tissue ELISA measurements (TNF-α, IL-6), immunohistochemical evaluations (IL-1β, TGF-β), and DNA comet assay will significantly contribute to filling the existing knowledge gap in the literature and revealing the hepatotoxic profile of DEP in more detail.

## 2. Materials and Methods

### 2.1. Chemicals

In the experiment, 99% pure diethyl phthalate (DEP; CAS No. 84-66-2; Sigma-Aldrich, Merck, KGaA, Darmstadt, Germany) was dissolved in corn oil and administered orally by gavage at doses of 100, 300, or 600 mg/kg/day for 21 days, with dosing volumes adjusted daily according to body weight.

### 2.2. Ethical Approval and Animal Model

The Dicle University, Sciences Research and Application Center Local Ethics Committee for Experimental Animals granted ethical permissions for our investigation (decision number: 14, date 25 September 2025).

In all, 28 female Wistar albino rats weighing 180–200 g and aged 8–10 weeks were employed in the investigation. The animals were divided into four equal groups of seven rats each at random. The animals were kept in a typical laboratory setting with a 12 h light/12 h dark cycle, 23 ± 5 °C, and 45% relative humidity for the duration of the study. They were given regular pellet meal and unlimited access to tap water. At the end of the experiment, all animals were anesthetized using a mixture of xylazine and ketamine hydrochloride. Blood samples for serum biochemical biomarkers were collected into a yellow biochemistry tube (BD Vacutainer^®^, Belliver Industrial Estate, Plymouth, UK) for the measurement of liver function tests (alanine aminotransferase (ALT), aspartate aminotransferase (AST), albumin (ALB), total bilirubin (TBIL), direct bilirubin (DBIL), cholesterol (CHOL), triglycerides (TRIG), and total protein (TP). The livers of each animal group were removed. A portion of the liver lobes was stored in a deep freezer at −80 °C (Binder GmbH, UF V 500, Tuttlingen, Germany) until inflammation analyses were performed. For additional histological analysis, samples from the remaining section were preserved in 10% neutral buffered formalin. In order to evaluate the amounts of inflammatory cytokines (IL-6, TNF-α), IL-1β, TGF-β, as well as histopathology (H&E and Masson’s trichrome staining), liver tissue was kept at −80 °C, as was the case for the Comet assay, to evaluate DNA damage. Our working groups were as follows:

Group 1 (Control): Received corn oil only (0.5 mL/day);
Group 2 (100 mg/kg/day): DEP in corn oil (dose adjusted daily to body weight);
Group 3 (300 mg/kg/day): DEP in corn oil (dose adjusted daily to body weight);
Group 4 (600 mg/kg/day): DEP in corn oil (dose adjusted daily to body weight).

It has been reported that the oral LD_50_ value of diethyl phthalate (DEP) is approximately 8600 mg/kg body weight. Previous studies have shown that oral DEP exposure up to 750 mg/kg/day does not lead to lethal effects [14]. Based on these data, the dose range of 100–600 mg/kg/day used in the study was determined to be non-lethal doses that are significantly below the LD_50_ but allow for the demonstration of biological and toxicological responses.

The selected dose range was structured to include low, medium, and relatively high exposure levels, allowing for the assessment of the dose–response relationship. The experimental exposure duration was designed to be consistent with the durations commonly applied in OECD subacute toxicity testing guidelines [15]. The aim of this dosing strategy is to evaluate the mechanistic and functional toxic effects of DEP under controlled experimental conditions, and no direct extrapolation to human exposure levels is intended. Instead, the selected doses establish a toxicologically relevant and experimentally justified dose range that allows for the investigation of DEP-induced biological effects within a non-lethal exposure range.

### 2.3. Analyses of Serum Biochemical Biomarkers

Blood samples from each animal were centrifuged at 1500 g for 10 min (Megafuge STPlus, Thermo Scientific, Waltham, MA, USA). Serum supernatants were collected into sterile tubes, and liver function tests (AST, ALT, TBIL, CHOL, TRIG, ALB, and TP) were analyzed on a biochemical autoanalyzer (Architect c8000; Abbott, Wiesbaden, Germany) using ultraviolet spectrophotometric, colorimetric, and enzymatic methods.

### 2.4. Histopathological Evaluation

In order to do our histological evaluations of the variations in liver tissue between the exposure groups, tissue specimens obtained at the conclusion of the trial were kept in a 10% solution of formaldehyde. After 72 h of exposure to formaldehyde, the tissues were rinsed under running water. Following a series of procedures to eliminate alcohol, they were washed in xylene, placed in paraffin, and finally formed into paraffin blocks. Five-micron-thick slices of rat liver paraffin blocks were placed on slides. Masson’s trichrome staining and Hematoxylin and Eosin were used to stain the sections. Following staining, the slices were rinsed with increasingly concentrated alcohol, cleaned with xylene, mounted on coverslips, and examined under a light microscope (Nikon Eclipse Si, Tokyo, Japan). Histopathological evaluation was performed by examining ten randomly selected microscopic fields per animal. Hepatocyte necrosis, cytoplasmic vacuolization, sinusoidal dilatation, and congestion in liver tissue injury were used to evaluate group differences. A scale of 0 to 3 was used to rate each criterion, with 0 denoting none, 1 mild, 2 moderate, and 3 severe. All evaluations were conducted in a blinded manner by two independent histologists who were unaware of the treatment groups [16,17].

### 2.5. Immunohistochemical Evaluation

Immunohistochemistry showed that IL-1β and TGF-β were present in liver tissue. Immunohistochemical staining uses the avidin-biotin peroxidase test. We made five-micrometer-thick sections of paraffin blocks on polylysine slides so that we could stain them. Xylene was used to remove the paraffin from the sections, and then a gradient of alcohol was used to keep them safe. Finally, distilled water was used for drying them out. Sections were heated with 5% citrate buffer in a 600 W microwave oven to restore the antigens, washed with phosphate-buffered saline (PBS), and then treated with 3% H_2_O_2_ to inhibit endogenous peroxidase activity. All subsequent procedures used the immunohistochemical staining kit (Thermo Fisher Scientific Inc., Waltham, MA, USA), and the process was carried out in an environment that prevented the tissue from drying out. Blocking serum was applied to PBS-washed sections and allowed to remain at room temperature for ten minutes in order to cover the regions outside of the antigenic sites. We kept the main antibodies for IL-1β (Proteintech, 26048-1-ap, 1:200, Wuhan, China) and TGF-β (Proteintech, Cat No. 21898-1 AP, 1:250) at 4 °C overnight. After that, the slices were treated with secondary antibodies that had biotin attached to them. After washing with PBS, the streptavidin-peroxidase mixture was applied. In order to reveal the immunoreactivities, the sections were next cleaned and treated with diaminobenzidine (DAB) (Dia-minobenzidine chromogen and substrate system, 125 mL from Thermo Fisher Scientific, Waltham, MA, USA). Then, they were washed again for five minutes with distilled water. The tissues were then put in a series of higher and higher concentrations of alcohol, xylene, and Entellan to keep air bubbles from forming. We looked at the areas that had been stained with immunohistochemistry under a light microscope and took pictures of them from ten randomly selected places per animal. The immunoreactivity of the markers in the histopathological images was examined using ImageJ software (version 1.54r, NIH, Bethesda, MD, USA). All analyses were performed in a blinded manner, and the evaluators were unaware of the treatment groups during quantification. The staining intensity was measured using a color threshold method with identical threshold settings applied to all images, and background staining was excluded prior to analysis. The outcomes were recorded and summarized on a per-animal basis for statistical evaluation [18].

### 2.6. Measurements of Inflammatory Cytokines (IL-6, TNF-α) in Rat Liver Tissue

Liver tissues stored at −80 °C were weighed on a precision balance and homogenized in phosphate-buffered saline (PBS, pH 7.2) at a 1/10 (weight/volume) ratio (Sigma-Aldrich Co., Bremen, Germany). Homogenates were centrifuged at 5000× *g* for 5 min at 4 °C (Thermo Scientific, USA), and the supernatants were collected. Interleukin-6 (IL-6); (Sun Red Bio, cat. no: 201-11-0136, Shanghai, China) and tumor necrosis factor-alpha (TNF-α); (Sun Red Bio, cat. no: 201-11-0765, Shanghai, China) levels were measured in the supernatant of liver tissue homogenates using commercial rat-specific ELISA kits according to the manufacturer’s instructions. Absorbance values were read at a wavelength of 450 nm using an ELISA reader (Rel Assay Diagnostics, Mega Tıp, Gaziantep, Turkey). Cytokine concentrations were calculated using standard curves provided by ELISA kits and expressed in pg/mL for IL-6 and ng/L for TNF-α according to the manufacturer’s validation. Reported values are concentrations measured in the supernatant of homogenized liver tissue at a constant ratio (1/10, *w*/*v*) and are expressed in accordance with the manufacturer’s recommended analysis and reporting format [19].

### 2.7. Determination of DNA Damage

Single-cell gel electrophoresis (SCGE), also known as the DNA comet assay, is a sensitive and reliable fluorescence microscopy technique for detecting DNA damage at the single-cell level. In this study, DNA damage in liver tissue was assessed using the neutral comet assay. Liver tissues were finely minced on ice using a sterile scalpel, and 2 mL of cold Ca^2+^- and Mg^2+^-free PBS was added. The suspension was homogenized on a magnetic stirrer at 500 rpm for 10 min, then allowed to settle for 10 min. The supernatant containing isolated cells was used for further analysis. Microscope slides were pre-coated with 0.5% normal melting point agarose and allowed to dry at room temperature. Subsequently, 100 µL of cell suspension was mixed with 1000 µL of 0.8% low melting point agarose at 37 °C, layered onto the coated slides, and covered with coverslips. Slides were solidified on ice at 4 °C for 5 min, after which coverslips were removed. Slides were then immersed in freshly prepared cold lysis solution (TBE buffer containing 25 g SDS) at 4 °C for 4 min. After lysis, slides were placed in a horizontal electrophoresis chamber containing fresh TBE electrophoresis buffer (54 g Tris, 27.5 g boric acid, 20 mL EDTA; pH 8.4) and incubated for 20 min to allow DNA unwinding. Electrophoresis was performed at 64 V and 250 mA for 2 min at room temperature. Following electrophoresis, slides were washed in distilled water (dH_2_O) for 5 min, neutralized, and stained with 50 µL ethidium bromide (1 µg/mL). All experimental procedures were conducted under low-light conditions to minimize artificial DNA damage. Slides were examined using an Olympus BX51 fluorescence microscope (Olympus Corporation, Tokyo, Japan) at 200× magnification. Each group consisted of seven rats. One slide was prepared for each rat in the group. In total, twenty-eight slides were prepared. 50 randomly selected cells per group of seven slides were analyzed using Comet Assay Software Project (CASP v1.2.2). DNA damage was quantified using the parameters head length, tail length, comet length, head DNA (%), tail DNA (%), tail moment, and olive tail moment. Cells displaying a comet-like tail were classified as DNA-damaged, whereas nuclei without a tail were considered undamaged [20].

### 2.8. Statistical Analysis

In our study, statistical evaluations were performed using SPSS version 21.0 (IBM Corp., Armonk, NY, USA), and the Shapiro–Wilk test was used to assess the normality of the data. Immunohistochemical quantitative data, serum biochemical parameters, inflammatory cytokine levels, DNA comet assay parameters, and mean ± standard deviation (SD) values were presented. One-way analysis of variance (ANOVA) and Tukey’s post hoc test were applied. Histopathological damage scores obtained from H&E staining were evaluated as ordinal data and analyzed using the Kruskal–Wallis test. Subsequently, post hoc analysis was performed using the multiple comparison test of Dunn. Statistical significance was defined at *p* values below 0.05.

## 3. Results

### 3.1. Analysis of Blood Samples

The results of our study’s serum biochemical parameters are given in Table 1. Statistical analysis between the groups revealed no significant differences in ALT, total bilirubin (TBIL), cholesterol (CHOL), triglycerides (TRIG), albumin (ALB), and total protein (TP) levels (*p* > 0.05), while a significant difference was found in AST and direct bilirubin (DBIL) levels (*p* < 0.05). AST activity was significantly increased, particularly in the groups treated with 100 and 300 mg/kg/day DEP, compared to the control group, while the 600 mg/kg/day group showed higher levels compared to the control group and similar levels to the other DEP groups. Direct bilirubin (DBIL) levels were increased in the 100 and 300 mg/kg/day groups compared to the control group, while they decreased to the control group level in the DEP 600 group.

### 3.2. Light Microscopic Results

**Figure 1:**
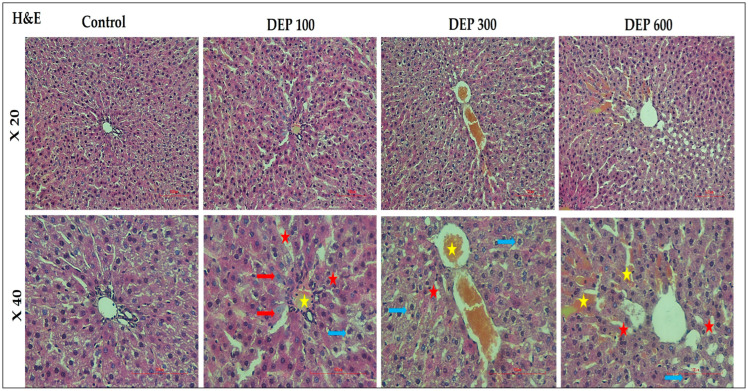
Light microscope findings in rat liver tissue (Nikon Eclipse Si, Tokyo, Japan, X200 and X400). HE-stained histological sections of the DEP 100, 300, and 600 groups show hepatocyte necrosis (red arrow), vacuolization in hepatocyte cytoplasm (blue arrow), sinusoidal dilation (red star), and congestion in the central veins (yellow star).

According to H&E staining results, the control group showed well-preserved lobular architecture of liver tissue, regular arrangement of hepatocyte cords, and intact sinusoids. Progressive histomorphological changes were observed in groups treated with DEP, depending on the dose. In the DEP 100 group, in addition to irregularity in hepatocyte cords and blurring of intercellular boundaries, hepatocyte necrosis (red arrow) and vacuolization in hepatocyte cytoplasm (blue arrow) were noted in some areas, and sinusoidal dilation (red star) was also observed. Congestion in the central veins (yellow star) was also noted. In the DEP 300 group, these findings became more pronounced, with increased disruption of the hepatocyte cord structure being particularly noteworthy. In the DEP 600 group, histological damage has reached the most advanced level; hepatocyte cords have lost their integrity in places. Congestion in the vessels has progressed, and the spread of these congested areas around the central vein is noteworthy. Another clearly identified finding is that sinusoidal dilatation has reached its highest level in this group (Figure 1).

**Figure 2:**
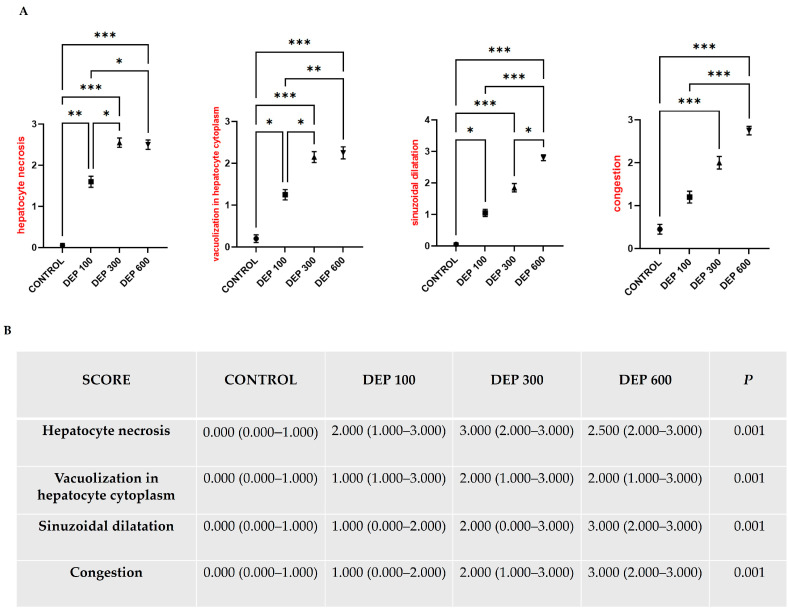
(**A**) Graphical representation of hepatocyte necrosis, vacuolization in hepatocyte cytoplasm, sinusoidal dilation, and congestion in the central veins in rat liver tissue groups. (**B**) Table displaying findings related to liver tissue. * *p* < 0.05, ** *p* < 0.01, *** *p* < 0.001. Values are presented as median (minimum–maximum) and were analyzed using the Kruskal–Wallis test, followed by Dunn’s post hoc test (Nikon Eclipse Si, Tokyo, Japan, X40).

In the scoring performed for the quantitative evaluation of H&E staining findings, the hepatocyte necrosis score was significantly elevated in the DEP 100 group compared to the control group (*p* < 0.01), while a significant increase was determined in the DEP 300 and DEP 600 groups compared to the control group (*p* < 0.001). Similarly, it was determined that the vacuolization score in hepatocyte cytoplasm and sinusoidal dilatation were significantly increased in the DEP 100 group compared to the control group (*p* < 0.05), and that this increase became more pronounced in the DEP 300 and DEP 600 groups and was statistically highly significant (*p* < 0.001). It was determined that the congestion score increased in parallel with the DEP dose increase, but no statistically significant difference was found in the DEP 100 group compared to the control group. In contrast, significant and marked increases were observed in the DEP 300 (*p* < 0.001) and DEP 600 groups (*p* < 0.001) compared to the control group (*p* > 0.05) (Figure 2A,B).

**Figure 3:**
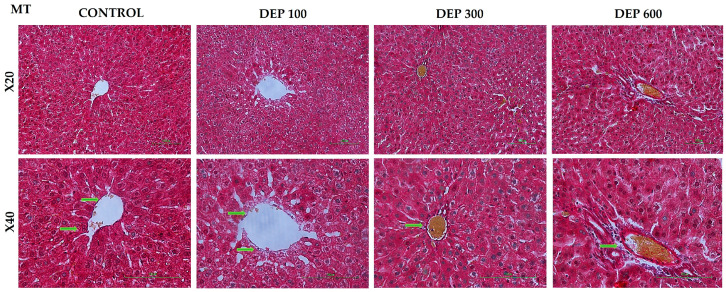
Light microscope findings in rat liver tissue. Control, DEP 100, 300, and 600 groups’ MT-stained histological section images. The pericentral region (green arrow) of the liver tissue is shown. (Nikon Eclipse Si, Tokyo, Japan, X200 and X400).

Collagen accumulation and fibrotic changes in liver tissue were evaluated using Masson’s trichrome staining. In the control group, collagen fibers showed a physiological distribution limited to the central vein and portal areas. In the groups treated with DEP, no significant and widespread increase in collagen was detected compared to the control group. In the DEP 100, DEP 300, and DEP 600 groups, collagen distribution around the central vein and in the perisinusoidal areas was generally similar to that in the control group, and no appearance suggestive of advanced fibrosis or fibrotic band formation was observed (Figure 3).

### 3.3. Immunohistochemical Results

**Figure 4:**
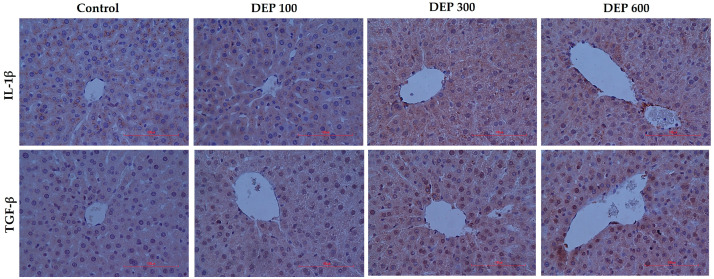
Immunohistochemical microscopic findings in experimental groups. Immunoreactivity density of IL-1β and TGF-β markers in liver tissue of all experimental groups (Nikon Eclipse Si, Tokyo, Japan, X400).

**Figure 5:**
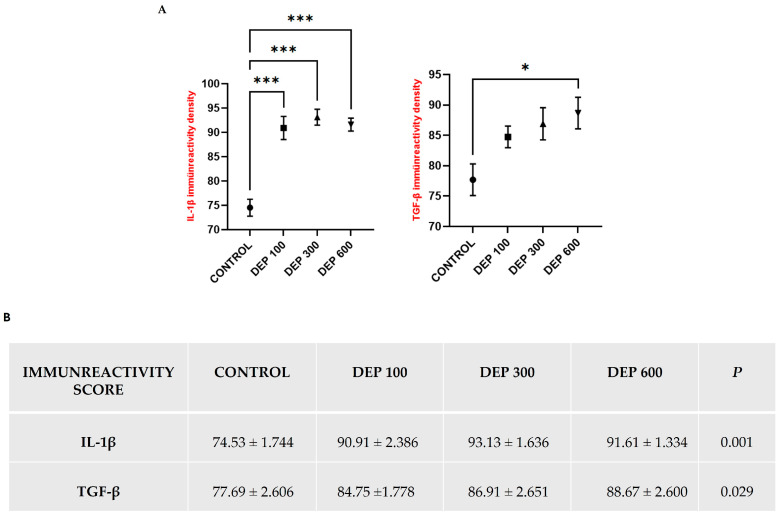
(**A**) Graphical representation of immunohistochemical findings. (**B**) Table representation of IL-1β and TGF-β marker expression in liver tissue across all experimental groups.* *p* < 0.05, ** *p* < 0.01, *** *p* < 0.001. Values are shown as mean ± SD and were analyzed using one-way ANOVA followed by Tukey’s post hoc test (Nikon Eclipse Si, Tokyo, Japan, X40).

When IL-1β expression is considered, it is noteworthy that this marker is particularly concentrated in hepatocytes adjacent to the central vein and sinusoidal areas, while in high-dose groups, it shows a more widespread distribution throughout the hepatocyte cytoplasm. According to quantitative analysis results, IL-1β immunoreactivity intensity was significantly increased in the DEP 100 group compared to the control group (*p* < 0.001), reached the highest level in the DEP 300 group (*p* < 0.001), and maintained this high level in the DEP 600 group (*p* < 0.001) (Figure 4 and Figure 5A,B).

TGF-β expression was again found to be concentrated around the central vein, but unlike IL-1β, it showed a more limited distribution in hepatocyte cytoplasm. In the immunohistochemical analysis of TGF-β, a gradual increase in immunoreactivity intensity was observed in the DEP-treated groups compared to the control group. Quantitative data show that TGF-β immunoreactivity intensity reached a statistically significant level, particularly in the DEP 600 group, compared to the control group (*p* < 0.05) (Figure 4 and Figure 5A,B).

### 3.4. Analysis of Inflammation Markers

In our study, the levels of IL-6 and TNF-α, inflammatory cytokines in liver tissue, were analyzed and presented in Table 2. According to the findings, DEP administration significantly increased the inflammatory response in female Wistar albino rats. The 100 mg/kg/day DEP group had considerably higher levels of IL-6 than the control group (*p* < 0.05), and the 300 and 600 mg/kg/day DEP groups had even higher levels (*p* < 0.001). Similarly, a significant increase in TNF-α levels was detected depending on DEP exposure; statistically higher values were observed in all DEP groups in contrast to the control group (*p* < 0.05). In addition, TNF-α levels were significantly increased in the 300 and 600 mg/kg/day DEP groups compared to the 100 mg/kg/day group. These findings indicate that DEP exposure can trigger a dose-dependent inflammatory process in liver tissue and increase the expression of pro-inflammatory cytokines.

### 3.5. DNA Comet Assay Analysis

Significant differences were identified between the groups for L-Head, L-Tail, L-Comet, Head-DNA, Tail-DNA, TM, and OTM, based on the statistical comparison conducted using the one-way ANOVA test (*p* < 0.001).

**Figure 6:**
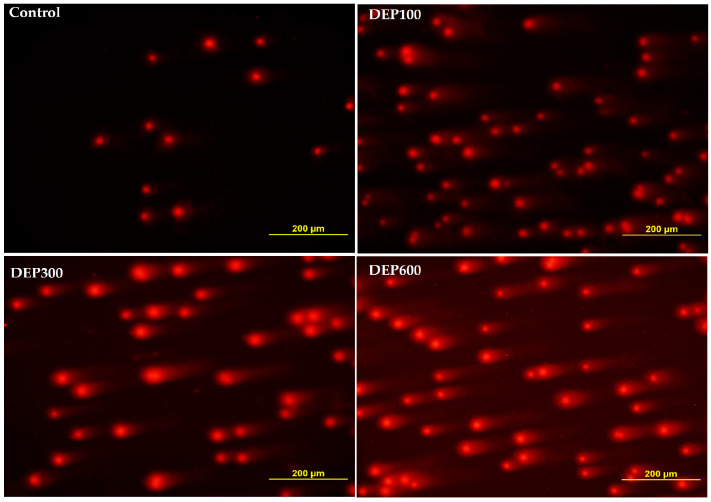
Comet images in liver tissue following diethyl phthalate exposure. The red dots represent liver cells. In the figure caption, the % DNA damage is numerically expressed in the tail (% Tail DNA). Control tail DNA 3.68%, DEP 100 tail DNA 10.26%, DEP 300 tail DNA 16.76%, DEP 600 tail DNA 25.64% (Ethidium bromide staining X200, Olympus, Japan).

**Figure 7:**
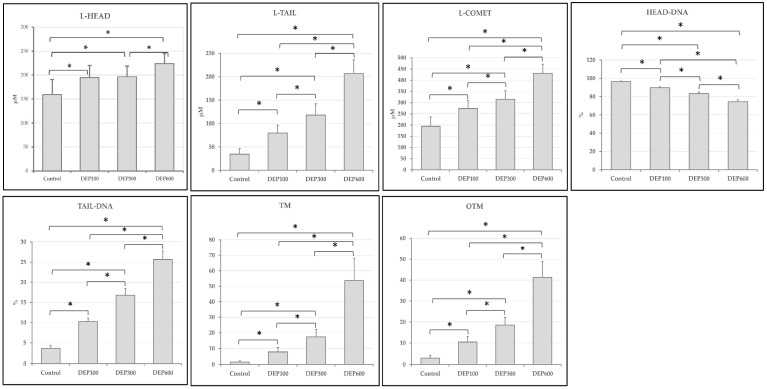
Comet assay results are presented in bar graphs, error bars, and within-group comparisons. * *p* < 0.001.

Comet assay analysis of the groups was performed according to the Post hoc Tukey test. The study revealed that L-Head levels were significantly increased in the DEP-100, DEP300, and DEP600 groups compared to the control group (*p* < 0.001). Furthermore, L-Head levels were significantly increased in the DEP600 group compared to the DEP300 group (*p* < 0.001). The study also revealed that L-Tail levels were significantly increased in the DEP-100, DEP300, and DEP600 groups compared to the control group (*p* < 0.001). Additionally, L-Tail levels were significantly increased in the DEP600 group compared to the DEP100 and DEP300 groups (*p* < 0.001). Finally, L-Tail levels were significantly increased in the DEP300 group compared to the DEP100 group (*p* < 0.001). The study revealed that L-Comet levels were significantly increased in the DEP-100, DEP300, and DEP600 groups compared to the control group (*p* < 0.001). Furthermore, L-Comet levels were significantly higher in the DEP600 group compared to the DEP100 and DEP300 groups (*p* < 0.001). Additionally, L-Comet levels were significantly higher in the DEP300 group compared to the DEP100 group (*p* < 0.001). The study also showed that Head-DNA levels were significantly decreased in the DEP-100, DEP300, and DEP600 groups compared to the control group (*p* < 0.001). Furthermore, Head-DNA levels were significantly lower in the DEP600 group compared to the DEP100 and DEP300 groups (*p* < 0.001). Finally, Head-DNA levels were significantly lower in the DEP300 group compared to the DEP100 group (*p* < 0.001). The study revealed that Tail-DNA levels were significantly increased in the DEP-100, DEP300, and DEP600 groups compared to the control group (*p* < 0.001). Furthermore, Tail-DNA levels were significantly higher in the DEP600 group compared to the DEP100 and DEP300 groups (*p* < 0.001). Similarly, Tail-DNA levels were significantly higher in the DEP300 group compared to the DEP100 group (*p* < 0.001). The study also showed that TM levels were significantly increased in the DEP-100, DEP300, and DEP600 groups compared to the control group (*p* < 0.001). Additionally, TM levels were significantly higher in the DEP600 group compared to the DEP100 and DEP300 groups (*p* < 0.001). Finally, TM levels were significantly higher in the DEP300 group compared to the DEP100 group (*p* < 0.001). The study revealed that OTM levels were significantly increased in the DEP-100, DEP-300, and DEP-600 groups compared to the control group (*p* < 0.001). Furthermore, OTM levels were significantly higher in the DEP-600 group compared to the DEP-100 and DEP-300 groups (*p* < 0.001). Similarly, OTM levels were significantly higher in the DEP-300 group compared to the DEP-100 group (*p* < 0.001). DNA damage findings in liver tissue are presented in Table 3 and Figure 6. The results for each group are presented collectively in Figure 7.

## 4. Discussion

DEP exposure in rats has been reported to cause significant damage to liver enzymes as well as morphological structure and biochemical parameters [11]. In our study, similar to previous studies, DEP exposure was found to have dose-dependent adverse effects on liver tissue. According to our results, dose-dependent DEP exposure resulted in structural damage in the liver, such as increased inflammatory cytokines, DNA damage, and disruption of hepatocyte cord structure, necrosis, vacuolization, sinusoidal dilation and vascular occlusion.

A cross-sectional study in adolescents in the United States reported significant but weak associations between urinary phthalate metabolites and liver function markers; decreased serum albumin and total protein levels and increased TBIL levels were reported to be associated with phthalate exposure [21]. Previous studies have reported that phthalate exposure during childhood and adolescence may be associated with changes in liver function markers, and in experimental animal models, it can lead to increased ALT and AST levels via oxidative stress pathways [22,23]. In another study, significant increases in AST and ALT levels were reported in male rabbits treated orally with DEP at a dose of 330 mg/kg/day for 30 days [24]. Another study conducted in humans evaluated the relationships between urinary phthalate metabolites (MEP and MEHP) and metabolic parameters; overweight individuals were reported to have lower total cholesterol and higher serum triglyceride levels compared to normal-weight individuals [11,25]. Despite these findings, studies specifically investigating the effects of DEP on serum biochemical parameters in both humans and experimental animals remain limited. In the present study, DEP exposure resulted in a significant increase in serum AST levels, while no statistically significant changes were observed in ALT and other routine biochemical parameters. AST is a transaminase found in the liver as well as in myocardium, skeletal muscle, kidney, and other tissues; therefore, increases in serum AST activity may not always reflect specific hepatocellular damage, while ALT is considered a more specific marker in the liver. In this study, DEP exposure led to significant increases in AST levels, while a similar increase in ALT and other routine serum biochemical parameters was not observed, suggesting that the increase in AST may be an isolated biochemical response [26]. Oxidative stress and mitochondrial dysfunction play a significant role among the underlying mechanisms of hepatotoxicity associated with phthalate exposure; phthalate metabolites can impair mitochondrial function by increasing reactive oxygen species (ROS) formation and causing lipid peroxidation, which can result in changes in biochemical markers [23]. Furthermore, the available data did not show a clear linear dose–response relationship in AST levels; this suggests that DEP-induced biochemical responses may reflect limited or threshold-level interactions and point to a response pattern different from the classical dose-proportional hepatotoxicity model. Overall, these results are consistent with experimental and preclinical studies reporting that biochemical changes due to phthalate exposure may manifest as limited and selective enzyme changes in early or short-term exposures, while more pronounced and widespread elevations of liver enzymes are mostly observed with longer exposure durations, higher doses, or in the presence of additional cellular stress factors.

A study conducted on adult rats showed that dibutyl phthalate (DBP) can lead to increased collagen deposition, profibrotic factor formation, inflammation, and toxicological effects via the ROS/TGF-β1 signaling pathway in liver tissue [27]. In a mouse study investigating the effect of diethylhexyl phthalate (DEHP) on macrophages in liver tissue, it was reported that the number of macrophages increased, the transcriptional levels of IL-1β and IL-6 in liver tissue rose, and consequently, IL-1β and TNF-α levels showed an upward trend [28]. A study on the effects of chronic diethylhexyl phthalate (DEHP) exposure on liver damage in chickens reported that pathological liver damage increased with increasing dose and duration, liver macrophage infiltration increased, IL-1β and TNF-α levels rose, and TGF-β levels decreased [29]. Rats given high doses (840 mg/kg/day) of DEP orally for 14 days were reported to experience histopathological damage in liver tissue compared to the control group, with significant structural changes observed, including central lobular necrosis, sinusoidal dilation, chronic venous congestion, and fatty degeneration (steatosis) [30]. A study investigating the effects of dibutyl phthalate on hepatic lipid metabolism presents evidence that it may increase hepatic lipid metabolism disorders and hepatic toxicity. Furthermore, histopathological evaluation with HE has been reported to reveal inflammatory factor accumulation, partial hepatocyte fusion, and focal necrosis [31]. A study investigating the sex-specific toxicity of DEP in Wistar rats over 6 months showed that long-term exposure could result in significant changes in liver histology, including degenerative changes in hepatocytes in the centrilobular region, disruption of liver structure in this area, and enlargement of sinusoidal spaces [32]. In our study, dose-dependent exposure to DEP in female Wistar albino rats led to significant hepatotoxic effects on the histopathology of liver tissue, as demonstrated by immunohistochemical and biochemical findings. Histomorphological analyses revealed irregularities in hepatocyte cords, cytoplasmic vacuolization, sinusoidal dilation, vascular congestion, and necrotic areas in the DEP-treated groups; these structural changes were observed to become more pronounced with increasing dose. These findings, particularly when considered alongside increased serum AST levels, suggest that in this subacute rat model, DEP exposure is associated with early hepatocellular alterations. Immunohistochemical analyses showed a significant increase in IL-1β expression in the central venous and sinusoidal regions, and quantitative analysis results indicated a dose-dependent increase in IL-1β immunoreactivity intensity. ELISA results also showed dose-dependent increases in IL-6 and TNF-α levels. This situation has shown that DEP exposure triggers a strong inflammatory response in the liver. In contrast, the limited increase in TGF-β immunoreactivity and the absence of prominent fibrotic bands in Masson’s trichrome staining indicate that structural fibrosis did not develop during the experimental period. Although a slight increase in TGF-β expression was observed at higher doses, the absence of accompanying histopathological findings of fibrosis suggests that this change may reflect activation of early profibrotic signaling pathways rather than mature fibrosis.

Studies on the effects of dose-dependent DEP exposure on liver tissue lack sufficient data in the literature regarding inflammatory processes and the consequences of DNA damage. Furthermore, high levels of pro-inflammatory biomarkers can cause damage to macromolecules such as DNA [33]. The comet test is a method used in scientific studies to determine the genotoxic potential of many chemicals [1]. Anderson et al. used the comet test method to investigate the effects of some phthalate esters on human cells. Their research using the comet test showed that these substances can damage DNA in human blood cells and eliminate the effects of DEHP in rat liver microsomes [34]. Phthalates are processed in the liver, which regulates the body’s energy metabolism; therefore, both long-term and short-term exposure to phthalates can cause liver damage [35]; however, experimental animal studies show that phthalates can cause hepatotoxicity [36]. Therefore, the present study investigated the genotoxicity of DEP exposure using the comet assay. In our study, dose-dependent DEP exposure to DNA damage in rat liver tissue was examined using the comet assay.

A study on environmental pollutants reported that high levels of pro-inflammatory cytokines were associated with increased levels of genotoxicity. The results of the study showed that interleukins were a possible determinant factor for the increase in tail DNA percentage, and that more complex modifications of DNA structure could also be catalyzed by the inflammatory process [33]. A study on arsenic exposure reported significant changes in tail length, head length, tail length, tail moment, and tail DNA percentage in rat kidney cells showing high levels of damage [37]. In the comet test results of our study, dose-dependent DEP exposure resulted in an increase in head length, tail length, comet length, tail DNA, tail moment, olive tail moment levels and a decrease in head DNA levels in liver tissue.

Our findings demonstrate that dose-dependent DNA damage in the current experimental rat model is accompanied by structural abnormalities, necrosis, and vacuolization in hepatocytes. These morphological distortions may be a cellular stress that could lead to the release of cytosolic enzymes such as AST into the circulation through weakening of cellular membrane integrity. The co-occurrence of genotoxic findings, such as increased serum AST levels and an increased comet appearance observed in liver tissue, suggests that DEP exposure is associated with biochemical and genetic damage at the hepatic level.

Our study findings demonstrate that increased DNA damage is associated with markers of inflammatory response, occurring concurrently with elevated serum AST and TGF-β levels. The observation of dose-dependent DEP exposure, along with increased pro-inflammatory cytokine levels, exacerbated DNA damage, and impaired hepatocyte structural integrity, suggests that inflammatory processes in the subacute rat model may be linked to genotoxic effects and liver-related biochemical changes.

The main limitations of this study include the failure to evaluate oxidative stress markers and related molecular parameters, which could directly reveal the underlying mechanisms of the observed biochemical and histopathological changes, thus limiting mechanistic interpretations. Furthermore, the use of only female animals prevents the analysis of sex-related differences and restricts the generalizability of the findings to both sexes. Additionally, the subacute experimental design of the study hinders the evaluation of the long-term effects of DEP exposure.

## 5. Conclusions

Our study findings demonstrate that subacute DEP exposure is associated with dose-dependent biochemical, inflammatory, genotoxic, and histopathological changes in the liver, rather than a clearly defined, linear dose-dependent hepatotoxic response. A significant increase in serum biochemical parameters, specifically AST levels, was observed. However, no concurrent elevations were detected in other routine liver function markers. At high exposure levels, the simultaneous presence of inflammatory responses with elevated pro-inflammatory cytokines (IL-6, TNF-α, and IL-1β), as well as histopathological changes such as hepatocellular necrosis, sinusoidal dilation, cytoplasmic vacuolization, and vascular congestion, suggests early or subclinical hepatic stress rather than severe hepatocellular damage. Furthermore, the increase in TGF-β expression in Masson trichrome staining in the absence of collagen deposition indicates activation of early profibrotic signaling pathways without progression to established fibrosis. Additionally, the detection of increased DNA damage by the Comet assay supports the idea that DEP has a genotoxic effect under subacute exposure conditions, but these findings do not definitively demonstrate permanent or irreversible genotoxicity. These results support a hepatotoxicity profile characterized by inflammatory, structural, and molecular damage rather than definitive functional liver failure. The current findings indicate that further studies involving environmentally relevant exposure levels, longer exposure durations, and toxicokinetic-based approaches are needed to reliably assess risks to human health.

## Figures and Tables

**Table 1:** Serum biochemical parameters obtained from female Wistar albino rats.

Parameters	Control	DEP, mg/kg Body Weight/Day			*p*
		100	300	600	
ALT (U/L)	76.86 ± 12.08	68.43 ± 8.20	68.71 ± 6.82	70.14 ± 9.46	0.313
AST (U/L)	96.43 ± 14.14 ^a^	133.43 ± 19.87 ^b^	164.43 ± 33.97 ^b^	132 ± 26.07 ^a.b^	0.001
TBIL (mg/dL)	0.11 ± 0.01	0.11 ± 0.01	0.12 ± 0.02	0.11 ± 0.01	0.087
DBIL (mg/dL)	0.01 ± 0.01	0.02 ± 0.01	0.02 ± 0.01	0.01 ± 0.00	0.037
CHOL (mg/dL)	70.43 ± 8.89	70.29 ± 6.90	72.86 ± 5.05	68.14 ± 4.49	0.619
TRIG (mg/dL)	50.71 ± 11.37	48.43 ± 6.97	52.71 ± 7.16	43.43 ± 20.71	0.168
ALB (g/dL)	3.93 ± 0.14	3.81 ± 0.13	3.89 ± 0.20	3.84 ± 0.10	0.493
TP (g/dL)	6.14 ± 0.21	6.04 ± 0.35	6.07 ± 0.37	6.06 ± 0.16	0.917

Values are shown as mean ± SD using ANOVA and Tukey tests. Different superscript letters within the same row indicate statistically significant differences between treatment groups (*p* < 0.05). ALT: Alanine Aminotransferase, AST: Aspartate Aminotransferase, TBIL: Total Bilirubin, DBIL: Direct Bilirubin, CHOL: Cholesterol, TRIG: Triglycerides, ALB: Albumin, and TP: Total Protein.

**Table 2:** Liver tissue inflammatory cytokine levels (IL-6 and TNF-α) in female Wistar albino rats.

Parameters	Control	DEP, mg/kg Body Weight/Day			*p*
		100	300	600	
IL-6 (pg/mL)	4.63 ± 0.07	4.92 ± 0.12 ^a^	5.13 ± 0.19 ^a.b^	5.23 ± 0.13 ^a.b^	0.001
TNF-α (ng/L)	3.68 ± 0.12	3.91 ± 0.11 ^a^	4.11 ± 0.10 ^a.b^	4.17 ± 0.09 ^a.b^	0.001

Values are shown as mean ± SD using ANOVA and Tukey tests. Different superscript letters within the same row indicate statistically significant differences between treatment groups. a: *p* < 0.05 compared to control, b: *p* < 0.05 compared to DEP100. IL-6: Interleukin-6; TNF-α: Tumor necrosis factor-alpha.

**Table 3:** Comet assay parameters of liver tissue.

Parameters	Control	DEP, mg/kg Body Weight/Day			*p*
		100	300	600	
L-Head	159.3 ± 31.52	194.52 ± 25.46	196.64 ± 21.89	223.44 ± 21.32	0.001
L-Tail	34.72 ± 11.66	79.58 ± 16.98	117.92 ± 24.18	206.8 ± 29.26	0.001
L-Comet	194.48 ± 41.01	273.9 ± 35.65	314.56 ± 39.04	430.24 ± 41.08	0.001
Head-DNA	96.32 ± 0.65	89.76 ± 0.89	83.24 ± 1.67	74.36 ± 2.15	0.001
Tail-DNA	3.68 ± 0.65	10.26 ± 0.88	16.76 ± 1.67	25.64 ± 2.15	0.001
Tail Moment	1.52 ± 0.74	7.92 ± 2.72	17.38 ± 4.73	53.74 ± 14.36	0.001
Olive Tail Moment	3 ± 1.26	10.4 ± 2.63	18.48 ± 3.63	41.28 ± 7.48	0.001

Values are shown as mean ± SD using ANOVA and Tukey tests.

## Data Availability

The data presented in this study are available upon request from the corresponding author.

## Abbreviations

The following abbreviations are used in this manuscript:

DEP	diethyl phthalate
ALT	alanine aminotransferase
AST	aspartate aminotransferase
ALB	albumin
TP	total protein
TBIL	total bilirubin
DBIL	direct bilirubin
CHOL	cholesterol
TRIG	triglycerides
IL-1β	interleukin-1 beta
IL-6	interleukin-6
TNF-α	tumor necrosis factor-alpha
TGF-β	transforming growth factor-beta
H&E	hematoxylin and eosin
MTS	Masson’s trichrome staining
ELISA	enzyme-linked immunosorbent assay
PBS	phosphate-buffered saline
